# Synergistic Activation of VDR-RXR Heterodimers by Vitamin D and Rexinoids in Human Kidney and Brain Cells

**DOI:** 10.3390/cells13221878

**Published:** 2024-11-14

**Authors:** Mobin Emran Doost, Jennifer Hong, Jennifer E. Broatch, Michael T. Applegate, Carl E. Wagner, Pamela A. Marshall, Peter W. Jurutka

**Affiliations:** School of Mathematical and Natural Sciences, Arizona State University, Glendale, AZ 85306, USA; medoost@asu.edu (M.E.D.); jhong55@asu.edu (J.H.); jennifer.broatch@asu.edu (J.E.B.); michaeltrentapplegate@msn.com (M.T.A.); carl.wagner@asu.edu (C.E.W.); peter.jurutka@asu.edu (P.W.J.)

**Keywords:** vitamin D receptor, vitamin D, RXR, rexinoids, synergism, polymorphisms

## Abstract

The active form of vitamin D, 1,25-dihydroxyvitamin D (1,25D), binds to the vitamin D receptor (VDR) with high affinity. The VDR then heterodimerizes with the retinoid X receptor (RXR) and associates with vitamin D response elements (VDREs) to regulate the transcription of target genes. Bexarotene (Bex) is an RXR ligand (rexinoid) developed to treat cutaneous T-cell lymphoma and is a putative therapeutic for other diseases. We postulate that VDR ligands (1,25D) and RXR ligands (Bex/analogs) can “synergize” to “super-activate” the VDR-RXR heterodimer. This “cross-talk” could allow disorders treated with high-dose Bex therapy (leading to significant adverse side effects) to instead be treated using both low-dose Bex and vitamin D. Thus, we designed experiments to examine the effect of both VDR and RXR ligands, alone and in combination, to activate VDR-RXR-mediated transcription. The goal was to determine if selected RXR-specific ligands can synergize with vitamin D to amplify RXR-VDR activity. The results demonstrate a synergistic effect with both Bex and 1,25D which could be further modulated by (1) the protein levels (or polymorphic version) of VDR present in the cell, (2) the concentration of the ligands, (3) the cellular “background” (e.g., brain cells versus kidney cells), (4) the nature of the VDRE platform, or (5) the type of rexinoid (Bex analogs). Our findings suggest that diseases that respond to treatment with either vitamin D, or with rexinoids, may be amenable to enhanced therapeutic potential by employing multi-ligand dosing via combinatorial therapy.

## 1. Introduction

### 1.1. Vitamin D, VDR, and VDREs

Vitamin D3 can be acquired either through dietary intake or synthesized in the skin by a process dependent on UV light, where 7-dehydrocholesterol is converted to vitamin D3. In the liver, vitamin D3 undergoes hydroxylation to 25-hydroxyvitamin D (25D), which is the primary circulating form commonly measured in clinical settings. The enzyme CYP27B1, primarily located in the kidneys but also present in epithelial, neural, and immune tissues, converts 25D into the biologically active hormone, 1,25-dihydroxyvitamin D (1,25D), which is transported in the bloodstream bound to the vitamin D-binding protein [1]. With roles including regulating calcium and phosphate levels in the body by regulating absorption and reabsorption, suppressing parathyroid hormone (PTH), and stimulating the production of bone-derived FGF23, vitamin D is a main player in calcium and phosphate homeostasis [1]. Apart from its main functions, vitamin D demonstrates additional roles such as detoxification [2], potential enhancement of cell lifespan [3], modulation of immune responses [4], and regulation of metabolism [5], and it can also strengthen blood vessels [6] and protect the body from autoimmune disorders [7]. Furthermore, 1,25D is associated with xenobiotic detoxification [8] and demonstrates anti-inflammatory [9,10,11] and anticancer properties [12,13]. These activities rely mostly on the conversion of circulating 25D to 1,25D within local tissues rather than on the circulating 1,25D produced by the kidneys [1].

The actions of 1,25D are primarily driven by the nuclear vitamin D receptor (VDR) to mediate its endocrine effects. This nuclear receptor exhibits a strong and specific binding affinity for 1,25D. The VDR is a 427 (or 424) amino acid protein in humans that comprises a zinc finger DNA-binding domain (DBD) that is situated at the N-terminus and a ligand-binding/heterodimerization domain (LBD) located at its C-terminus [14]. The C-terminal region of the VDR protein is composed of at least 12 alpha-helices that facilitate the binding of 1,25D to VDR and the interaction between VDR and its partner, retinoid X receptor (RXR), another member of the nuclear receptor family, as well as coactivators. Once 1,25D binds to the VDR, VDR releases corepressors, forms a heterodimer with RXR, and undergoes a structural alteration in its helix 12, aiding in the recruitment of coactivators. The VDR-RXR interaction occurs primarily at helices 9 and 10 of the VDR protein, along with the loop region between helices 8 and 9. In addition, interactions between coactivators and VDR have been identified predominantly within helices 3, 5, and 12 [1]. The combined zinc finger binding domains of the VDR-RXR heterocomplex identify and attach to vitamin D response elements (VDREs) on DNA. VDREs are associated with numerous genes across various physiological systems, such as with hFGF23 (involved in renal phosphate reabsorption) or hCYP3A4 (associated with xenobiotic detoxification) [1].

### 1.2. Retinoid X Receptor

The retinoid X receptor (RXR) [15] is part of the nuclear receptor superfamily, functioning as a ligand-dependent transcription factor. The RXR exists in three isoforms: RXRɑ, RXRβ, and RXRγ [16]. Although RXRs are distributed throughout the body, RXRɑ is predominantly expressed in the kidneys, lungs, intestines, and muscles. The importance of RXR lies in its ability to form homodimers or heterodimers with other crucial receptors, thereby regulating gene expression and impacting cellular development and metabolic signaling [17]. RXR serves as a vital component in several receptor pathways, including the liver X receptor (LXR), the thyroid hormone receptor (TR), the vitamin D receptor (VDR), and the retinoic acid receptor (RAR), among others. These nuclear receptors primarily regulate gene expression by modulating transcription, typically in response to the presence of a ligand associated with the receptor and its obligatory partner [16,18].

RAR, VDR, and TR form heterodimers with RXR to bind to their hormone response elements (HREs). When RXR is bound to its native 9-cis-retinoic acid (9-cis-RA) agonist ligand, it forms an RXR homodimer that subsequently binds to the RXR responsive element (RXRE). However, when RXR pairs with other nuclear receptors as a heterodimer, it does so with or without a ligand in the RXR LBD (ligand-binding domain). Early evidence showed that RXR remains unoccupied in the RXR-VDR heterodimer, although there is no universal agreement on this in all cases. Alternatively, there are also suggestions that RXR may be bound to a ligand when it forms a heterodimer with VDR and with other receptors such as LXR [19]. This distinction has categorized RXR–heterodimer complexes into two classes: non-permissive and permissive. Non-permissive heterodimers can solely be activated by the ligand of the RXR partner receptor, while permissive heterodimers can be activated either by an RXR ligand or by a ligand specific to the partner receptor [16,18]. Nevertheless, RXR is deemed to have a significant role due to its function as a central regulator among nuclear receptors because it participates in the formation of heterodimer complexes with other receptors, assisting with DNA binding to their respective response elements [20].

### 1.3. Rexinoids and Bexarotene

Due to the function of RXR as a central regulator, the development of agonists targeting the RXR has been of considerable interest. Selective RXR ligands, also known as rexinoids (or selective nuclear receptor modulators, SNuRMs), offer a pharmaceutical target for medicinal purposes. By specifically activating RXR instead of other hormone receptors, these rexinoids offer potential chemotherapeutic benefits for many human cancers without harmful or adverse effects that can be associated with activating other pathways [20]. One highly specific RXR agonist is commonly known as bexarotene (Bex) which was developed to mimic the actions of the molecule 9-cis retinoic acid (RA). Bex is an FDA-approved drug indicated for the treatment of cutaneous T-cell lymphoma (CTCL) among other conditions. Analogs of Bex were further developed and have demonstrated similar specific RXR agonistic activity [18,21,22,23]. Although Bex is primarily used for the treatment of CTCL, it has also been investigated as a possible therapy for colon and breast cancers [24,25] and an off-label therapy for lung cancer [26]. However, Bex therapy also confers significant limitations due to its well-documented side effects. The side effects include hypothyroidism, cutaneous toxicity, and hyperlipidemia [27]. Furthermore, Bex can significantly increase the levels of serum lipids, including cholesterol and triglycerides, which can increase the risk of cardiovascular issues such as heart disease and stroke [27].

Therefore, there is a strong incentive to create new RXR-selective agonists or to develop novel dosing and/or combinatorial therapeutic strategies to mitigate these rexinoid-dependent side effects. Moreover, because some of the same diseases such as cancer are thought to be amenable to treatment by both rexinoids and vitamin D, we explore the relationship between VDR and RXR ligands and their potential cross-talk [28], as well as examine the presence of a multi-ligand pathway that may facilitate synergistic activation of VDR signaling. Utilizing novel rexinoids as well as vitamin D dosing, we analyze the potential for these two ligands to act synergistically to enhance VDR-RXR-mediated transcription (e.g., synergy is defined as transcriptional activation seen when both 1,25D and a rexinoid are present that is above the sum of individual activities alone).

## 2. Materials and Methods

### 2.1. Mammalian Cell Culture

Human embryonic kidney (HEK-293) cells or human glial brain (U87) cells were used for the indicated assays. The cells were obtained from the American Type Culture Collection (ATCC, Manassas, VA, USA, Catalog #CRL-1573 and Catalog #HTB-14). The cells were grown in Dulbecco’s Modified Eagle Medium (DMEM; Cytiva, Marlborough, MA, USA), grown with a high glucose concentration, with L-glutamine, sodium pyruvate, and phenol red, supplemented with 10% fetal bovine serum (FBS) and 100 U/mL penicillin/streptomycin (P/S), in humidified conditions at 37 °C with 5% carbon dioxide (CO_2_) and plated and passaged with high-glucose media as described above.

### 2.2. Luciferase Assay

#### 2.2.1. Plating and Transfection of Human Embryonic Kidney 293 and U87 Cell Lines

At time zero, the cells were plated into 24-well cell culture plates at a cell density of 47,000 to 60,000 cells/well 18–24 h before transient transfection (Fisher Scientific, Waltham, MA, USA). The appropriate plasmid DNAs were introduced to the cells using a polyethyleneimine (PEI) transfection reagent (Fisher Scientific). The cells in each individual well received 1.25 μL/well of PEI reagent, 20 ng/well of pRL-null (the *Renilla* control plasmid), and 250 ng of a Firefly luciferase plasmid which included either the XDR3, PER6, or pFR-Luc vector.

The additional plasmids for each experiment are as follows: For experiments where exogenous VDR was added, 50 ng of pSG5-hVDR (M4 VDR polymorphism) was used. Two distinct VDREs were also utilized, depending on the experiment: XDR3-Luc and PER6 (as described by Dampf Stone et al., 2015) [29]. For the exogenous VDR M1 polymorphism, 50 ng of the plasmid pSG5-M1-hVDR (M1 VDR polymorphism) was employed. For the effects of two different concentrations of 1,25D the Firefly luciferase plasmid XDR3-Luc was used. For the mammalian 2-hybrid (M2H) system, we used pFR-Luc as the Firefly luciferase plasmid along with 0.2 ng of the pCMV-VDR binding domain vector (BD) and 0.2 ng of the pCMV-RXR activation domain (AD). For experiments in U87 brain cells, the Firefly luciferase plasmid XDR3-Luc with 50 ng of pSG5-hVDR was employed.

The original human VDR expression vector (pSG5-hVDR-M4) was created as described previously [30] and is employed in the current study. A common polymorphic variant that contains three additional amino acids at the N-terminus was generated via the method outlined in the Chameleon Double-Stranded, Site-Directed Mutagenesis Kit (Stratagene, La Jolla, CA, USA) utilizing double-stranded hVDR cDNA which contains amino acids 4–427, designated M4; see Baker et al. [14]. This insertional construct created from the M4 hVDR (amino acids 4–427) cDNA includes all 427 residues of the hVDR (and is designated M1). Additional details are in Jurutka et al. [31]. The plasmids used for the mammalian two-hybrid system, as well as the VDRE-containing plasmids, were prepared as described in Jurutka et al. [32].

#### 2.2.2. Treatment of the Cells with Various RXR Analogs and/or 1,25D at Different Concentrations, Cellular Lysis, and Luciferase Assay

After 22–24 h of transfection, the cells were dosed with the negative (vehicle) ethanol (EtOH) control, vitamin D, and/or the appropriate RXR agonist. All the compounds were solubilized in ethanol to acquire the targeted concentration of vitamin D and rexinoid. The concentrations ranged between 1 and 10 nM, as indicated in the various data figures.

Rexinoids were synthesized in-house, according to published procedures [18,20,33], except **CD3254**, which was purchased from Tocris (Bio-Techne Corporation, Minneapolis, MN, USA).

After 18 to 24 h of treatment with the appropriate ligands as indicated, the cells were lysed in 1X passive lysis buffer (Promega Corporation, Madison, WI, USA). The whole-cell lysates were analyzed for both Firefly and *Renilla* luciferase using a Dual-Luciferase Reporter Assay Kit (Promega Corp., Madison, WI, USA) according to the manufacturer’s protocols (Promega) in a Sirius FB12 luminometer (Berthold Detection Systems, Pforzheim, Germany). In order to account for cytotoxicity, cell death, and transfection efficiency from the ligand treatment, the data obtained were normalized by dividing the Firefly luciferase luminescence by the *Renilla* luciferase luminescence [34]. Any cytotoxicity of the compounds would decrease *Renilla* luminescence and allow for an internal control, although previous analysis of the compounds demonstrated no discernable cytotoxicity at the concentrations utilized in this study [18,20,33]. The activity of the reporter gene was measured in comparison to the reference compound 1,25D set to 100%.

**Figure 1:**
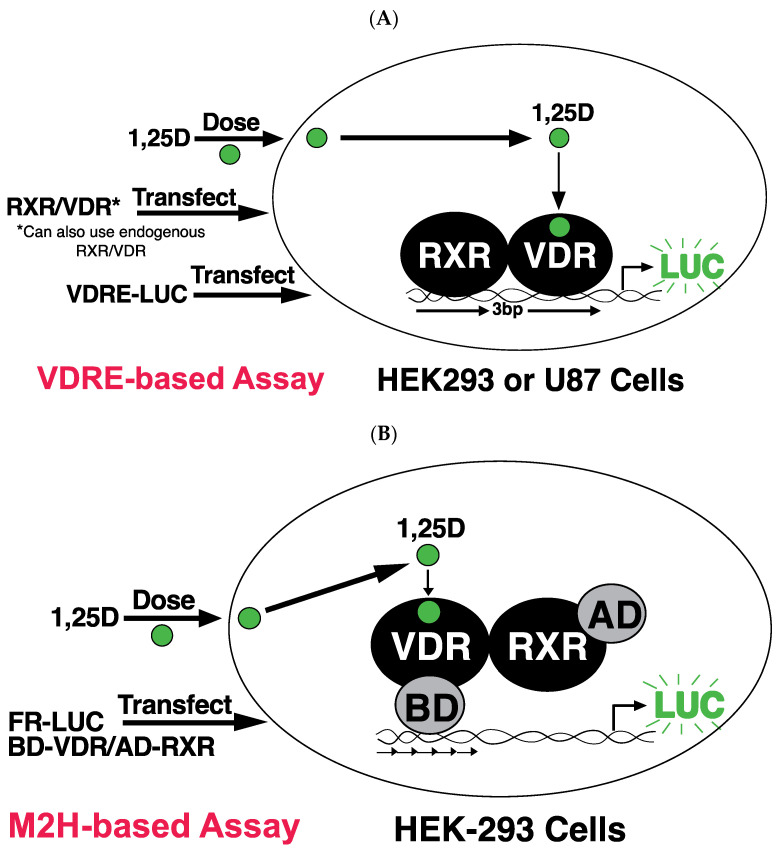
Schematic of assays used in this study. (**A**) The VDRE (XDR3 or PER6) assay: The VDRE assay employed in this study involved treating HEK-293 or U87 cells with ethanol (as a negative control), 1,25D (as a positive control), and RXR ligands (such as bexarotene or analogs), either individually or in combination with 1,25D. The extent of VDRE-mediated transcriptional activation, using direct repeat-3 as depicted in the figure, or another class of VDREs with an everted repeat (PER6), was assessed using light-based luciferase assays. For the experiments with exogenous VDR, cells were additionally transfected with VDR. (**B**) The mammalian 2-hybrid (M2H) assay: The M2H assay employed in this study involved treating HEK-293 cells with ethanol (as a negative control), 1,25D (as a positive control), and RXR ligands (such as bexarotene or analogs), either individually or in combination with 1,25D. The extent of M2H-mediated transcriptional activation was assessed using light-based luciferase assays.

Schematic of VDRE and Mammalian Two Hybrid (M2H) Assay are outlined in Figure 1. The VDRE assay determines the amount of transcriptional activity from a specific vitamin D responsive element with either endogenous or added (exogenous) VDR, in response to addition of 1,25D, or rexinoid or both. The M2H assay determines the amount of heterodimerization of RXR and VDR a given ligand stimulates.

Graphs represent mean and standard error. Replications were at least six samples per replication and at least three separate trials of each assay. Synergism was calculated as [VDRE or M2H activity of 1,25D+rexinoid]/([VDRE or M2H activity of 1,25D alone] + [VDRE or M2H activity of rexinoid alone] for each individual concentration of vitamin D.

### 2.3. Compound Properties and Principal Component Analysis (PCA) of Rexinoids

#### 2.3.1. Determination of Physiochemical Characteristics

The physiochemical properties of the selected rexinoids were determined and compared to each other via principal component analysis, a method used to reduce dimensions in a dataset to their principal component [35]. DataWarrior was used to determine HBA (hydrogen bond acceptor), HBD (hydrogen bond donor), TPSA (topological polar surface area), LogS, and cLogP [36]. Calculations of LogP and water solubility were performed using SwissADME [37]. cLogD was calculated using Chemaxon software Version 24.1.0 (Chemaxon, San Diego, CA, USA). The CNS MPO (Central Nervous System Multiparameter Optimization) Score was calculated using [38]. Physiochemical data were used to calculate violations of Lipinski’s Rules [39].

#### 2.3.2. Principal Component Analysis

Clustvis was utilized to perform PCA (principal component analysis) as indicated using physiochemical characteristics of the compounds [40].

### 2.4. Statistical Analysis

All other statistical analyses were performed in Microsoft Excel and SAS V 9.4 [41] where error bars represent the mean +/− the standard error of the mean. Statistical significance was determined at the overall α = 0.05 significance level, and all presented *p*-values were adjusted for multiple testing. Experimental data were analyzed with separate ANOVAs of treatments versus the respective controls. For initial XDR3 and PER6 experiments with multiple rexinoids, Dunnett’s method was employed post hoc to adjust for the overall family-wise Type I error for pair-wise comparisons of the six treatment groups versus the control. Since all pair-wise comparisons, specifically treatment-to-treatment comparisons, were not desired, the selection of Dunnett’s method avoids unnecessary comparisons and drastically reduces the number of comparisons from 21 to 6, resulting in a more powerful test. For experimental data presented inthe remainder of the experiments, the Tukey–Kramer method allowed for appropriate pair-wise comparisons of treatments and controls. The Tukey–Kramer method was a more powerful choice (versus Dunnett’s method) given the limited number of comparisons in experimental data from figures only utilizing two rexinoids. For experimental data presented in transcriptional activation tables, post hoc comparisons were adjusted for multiple testing using a Bonferroni correction for the specific comparisons identified a priori.

## 3. Results

### 3.1. Rexinoids Utilized in This Study and Rationale for Use

**Figure 2:**
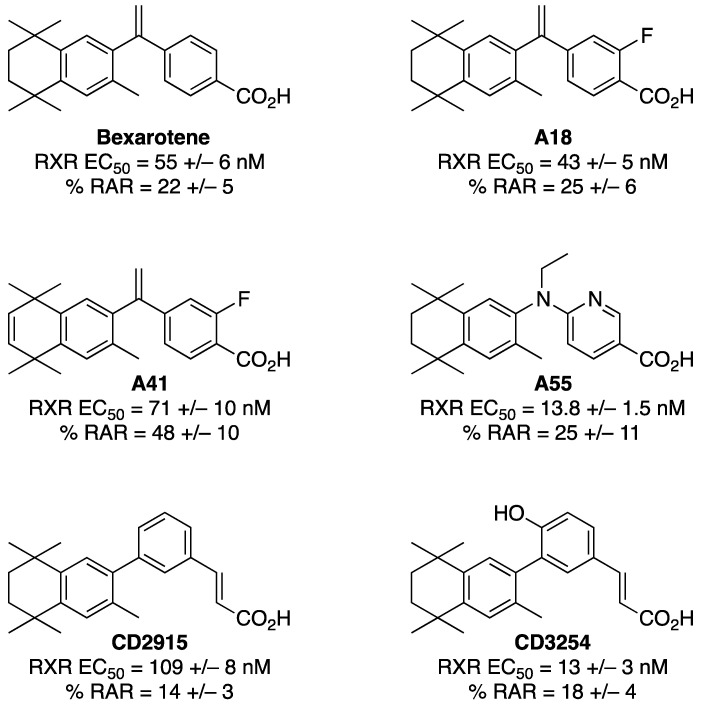
Structures and characteristics of bexarotene and additional rexinoids used in this study [18,20,33].

The rexinoids chosen for this analysis comprise a wide array of Bex analogs to compare and contrast effects for compounds exhibiting a few similar receptor activities—EC_50_ value for RXR activation and % RAR activation—but different structural features as well as biological activity. We selected these specific compounds as an initial array of rexinoids to test, versus the FDA-approved bexarotene standard, because across several of our prior studies, we have observed many rexinoids of similar potency as bexarotene at the RXR to exert markedly different gene expression, PK, and side effect profiles. Because RXR is involved in several transcriptional pathways regulated by other nuclear receptors, different rexinoid structures not only result in different gene expression profiles—and thus impact observed side effects—but also different PK characteristics, since various rexinoid structural features profoundly impact different physical properties such as acidity and oral absorption, distribution, metabolism, and plasma concentrations. Thus, we selected a variety of rexinoids from our prior studies that we have observed to possess a similar RXR potency as bexarotene, but different biological activities (as elaborated below), in a first survey to determine which rexinoids might be most active and appropriate to start with, as a basis, to develop and optimize in future studies. Figure 2 summarizes RXR and RAR activities reported for these compounds.

These rexinoids, along with the bexarotene standard, comprise a wide array of cellular activities, prompting us to further investigate their potential to synergize with vitamin D. In terms of structural features, the six rexinoids in the study include the FDA-approved cancer drug, bexarotene, two rexinoids (A18 and A41) possessing the bexarotene carbon skeleton, two acrylic acid rexinoids—CD2915 [42] and **CD3254** [20,43]—which have been well documented in the literature, and A55 which is an example of a diaryl amine rexinoid [18]. A55 is more effective than bexarotene in breast and lung cancer mouse models and also exhibits lower plasma lipid side effects than bexarotene in those mouse models [44]. The A18 compound differs from bexarotene only by the addition of a fluorine atom adjacent to the carboxylic acid functional group, but it retains a very similar EC_50_ value for the activation of RXR and RAR crossover compared to bexarotene. The A41 compound, in addition to possessing a fluorine atom adjacent to the carboxylic acid group on Bex, exhibits an unsaturation in the aliphatic ring system of bexarotene which makes it more structurally rigid. Treating Sprague Dawley rats with A41 increases plasma lipid side effects compared to bexarotene dosed at 100 mg/kg after 24 h [45]. For the two acrylic acid compounds, the structural difference in substituting a hydroxyl group in **CD3254** for an aromatic hydrogen atom in CD2915 results in an EC_50_ value for the activation of RXR that is an order of magnitude lower for **CD3254** than CD2915. We were therefore interested in probing the effects of this array of rexinoids exhibiting diverse structural features that contribute to notable differences for in vitro and in vivo activities.

### 3.2. Effect ofVDRE DNA Platforms, VDR Concentrations, and RXR Analogs

In the current study, we designed experiments to examine the effect of both VDR ligands (1,25D) and RXR ligands (Bex/analogs), alone and in combination, to activate VDR-RXR-mediated transcriptional activation employing multiple VDRE DNA platforms. The assay system was composed of human embryonic kidney (HEK-293) cells that were transfected with a luciferase reporter gene. The conditions of this assay were varied to include different VDREs and ligands, as well as increasing amounts of VDR protein, to determine if select rexinoids can synergize with vitamin D to produce amplified RXR-VDR heterodimer activity. RXR levels were kept constant with endogenous RXR in the HEK-293 cells to ensure biological levels and appropriate activation by RXR and VDR ligands [46].

**Figure 3:**
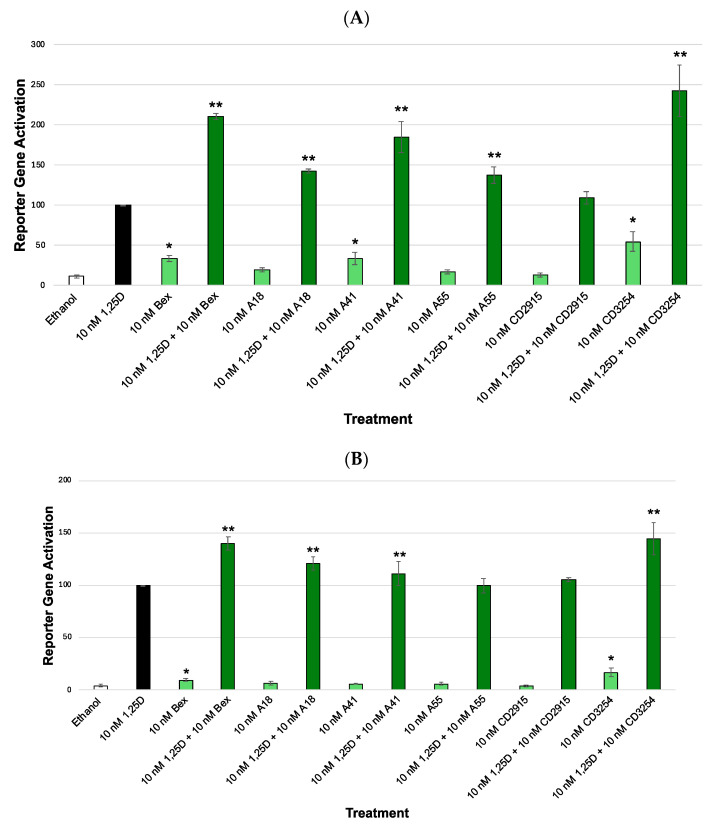
VDR and RXR XDR3 VDRE transcriptional activation. (**A**). XDR3 assay with endogenous VDR. Biological evaluation of 1,25D and bexarotene/analogs via a VDR-RXR (XDR3) VDRE–luciferase-based system in HEK-293 cells with endogenous VDR. All compounds were dosed at 10 nM. (**B**). XDR3 assay with exogenous VDR. Biological evaluation of 1,25D and bexarotene/analogs via a VDR-RXR (XDR3) VDRE–luciferase-based system in HEK-293 cells with overexpressed VDR (M4 polymorphism). The treatment groups are compared to the positive control 1,25D that was set to 100%, using an ANOVA with post hoc Dunnett’s method corrected *t*-tests. All compounds were dosed at 10 nM. An asterisk (*) indicates a statistically significant difference for the rexinoid treatment compared to the ethanol control (*p* < 0.05). A double asterisk (**) indicates a statistically significant difference between the rexinoid + 1,25D treatment and the 1,25D control (*p* < 0.05).

**Figure 4:**
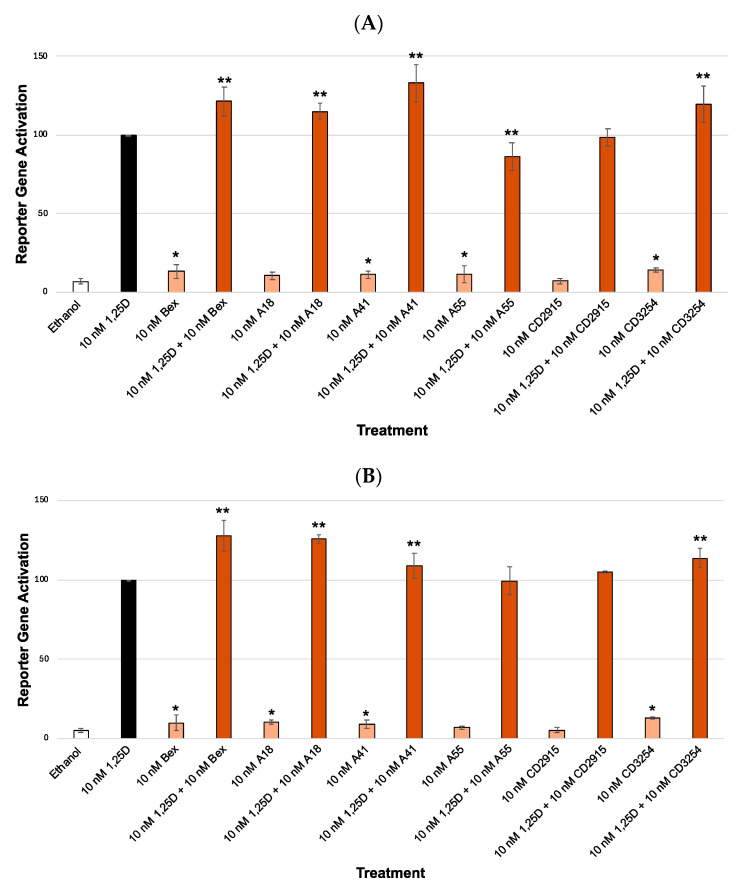
VDR and RXR PER6 VDRE transcriptional activation. (**A**). PER6 assay with endogenous VDR. Biological evaluation of 1,25D and bexarotene/analogs via a VDR-RXR (PER6) VDRE–luciferase-based system in HEK-293 cells with endogenous VDR. The treatment groups are compared to the positive control 1,25D that was set to 100%. All compounds were dosed at 10 nM. (**B**). PER6 with exogenous VDR. Biological evaluation of 1,25D and bexarotene/analogs via a VDR-RXR (PER6) VDRE–luciferase-based system in HEK-293 cells with overexpressed VDR (M4 polymorphism). The treatment groups are compared to the positive control 1,25D that was set to 100%, using an ANOVA with post hoc Dunnett’s method corrected *t*-tests. All compounds were dosed at 10 nM. An asterisk (*) indicates a statistically significant difference for the rexinoid treatment compared to the ethanol control (*p* < 0.05). A double asterisk (**) indicates a statistically significant difference between the rexinoid + 1,25D treatment and the 1,25D control (*p* < 0.05).

Our results indicate that the direct repeat VDRE (XDR3) (Figure 3A,B) possessed higher synergism than the everted repeated VDRE (PER6) (Figure 4A,B) when the cells were treated with 1,25D and/or Bex. For example, using the XDR3 VDRE with Bex and endogenous VDR (Figure 3A, bar 4), the amount of synergism was 90% greater compared to the PER6 VDRE with Bex and endogenous VDR (Figure 4A, bar 4). In addition, with the XDR3 VDRE with Bex and exogenous VDR (Figure 3B, bar 4), the amount of synergism was 12% greater compared to the PER6 VDRE with Bex and exogenous VDR (Figure 4B, bar 4). Moreover, in the presence of the XDR3 VDRE, the endogenous VDR (Figure 3A) produced much higher synergism when compared to cells transfected with extra (exogenous) VDR cDNA (Figure 3B). For example, using the XDR3 VDRE with Bex and endogenous VDR (Figure 3A, bar 4) the amount of synergism was 71% greater compared with the XDR3 VDRE with Bex and exogenous VDR (Figure 3B, bar 4). No major differences were observed in PER6 activation when endogenous versus exogenous VDR was assessed (Figure 4A,B). For example, when using the PER6 VDRE with Bex and endogenous VDR (Figure 4A, bar 4), the amount of synergism was only 6% less compared with the PER6 VDRE with Bex and exogenous VDR (Figure 4B, bar 4).

In addition to Bex, structurally diverse rexinoids (Figure 2) were evaluated. The results of the analog assays revealed that **CD3254** displayed the highest synergism with 1,25D, even greater than the positive control of Bex in the XDR3 assay primarily with endogenous VDR (Figure 3A) compared to the other analogs in their respective VDRE assays. Using the XDR3 VDRE and endogenous VDR (Figure 3A, bar 14), the amount of synergism of **CD3254** was 32% greater compared to Bex (Figure 3A, bar 4). In the XDR3 VDRE and exogenous (extra) VDR (Figure 3B, bar 14), the amount of synergism of **CD3254** was 4% greater compared to Bex (Figure 3B, bar 4). These differences were more muted (but some were still statistically significant) when employing the PER6 VDRE. For example, with the PER6 VDRE and endogenous VDR (Figure 4A, bar 14), the amount of synergism of **CD3254** was actually 2% less compared to Bex (Figure 4A, bar 4). However, in the PER6 VDRE and endogenous VDR (Figure 4A), analog A41 possessed a greater amount of synergism than Bex and **CD3254**. In the PER6 VDRE and endogenous VDR experiment (Figure 4A, bar 8), the amount of synergism of A41 was 12% greater compared to Bex (Figure 4A, bar 4). Finally, in the PER6 VDRE and exogenous (extra) VDR (Figure 4B, bar 14), the amount of synergism of **CD3254** was 14% less compared to Bex (Figure 4B, bar 4). None of the selected analogs were able to synergize to a greater extent than Bex in the PER6 VDRE and exogenous (extra) VDR (Figure 4B) including **CD3254**. Therefore, **CD3254** revealed the highest synergism with the XDR3 VDRE and endogenous VDR (Figure 3A, bar 14).

Furthermore, it can be noted from Figure 3 and Figure 4 that the cells that were treated with only Bex or with some of the rexinoid analogs (alone, without 1,25D) displayed observable activation compared to the ethanol (vehicle) control (*, *p* < 0.05 for those rexinoids with statistically significant enhancement of VDR-RXR). This important observation indicates that VDR is not solely a non-permissive heterodimer, as non-permissive heterodimers cannot be activated by the rexinoid ligand alone. This suggests that there are some instances of rexinoid-driven RXR-VDR heterodimerization and transcriptional activation that may occur in the absence of any 1,25D ligand, but this activation is minor compared to the activation that is elicited by the primary (1,25D) ligand.

### 3.3. Effect of VDR Polymorphisms

The vitamin D receptor (VDR) is the mediator of all biological actions of vitamin D. The *VDR* gene is located on chromosome 12 (12q13.11), and thus far, 900 allelic variants have been discovered. The most commonly studied *VDR* gene polymorphisms with the most research are ApaI (rs7975232), BsmI (rs1544410), Taql (rs731236), and Fokl (rs10735810). ApaI, TaqI, and BsmI are all silent genetic variants (found in the non-coding region of the gene) that increase the stability of the mRNA. The FokI locus is involved in the translation of the active VDR protein and consists of a polymorphism that is located on exon 2 on the 5’ region of the gene [47]. This polymorphism in human VDR results in two allelic isoforms (f/M1 and F/M4), with the M4 resulting in a protein shortened by three amino acids (424 vs. 427 amino acids). The human VDR containing an f/M1 (also known as ff) single-nucleotide polymorphism (SNP), which initiates transcription at an ATG start site three amino acids upstream from the F/M4 variant start site (also known as FF), is proposed to be less effective in mediating VDRE-dependent transcription in comparison to the F/M4 variant which lacks the first three N terminal amino acids. Differences in the isoforms of VDR are thought to impact VDR transcriptional activity and can modulate increased risk in vitamin D-related diseases [48]. These genetic variants have been associated with susceptibility to chronic diseases such as type 2 diabetes, autoimmune diseases, cancer, cardiovascular alterations, rheumatic arthritis, and metabolic bone diseases [47]. However, the influence of the common VDR M1/M4 variant on the synergism of rexinoids and vitamin D activity is unknown.

**Figure 5:**
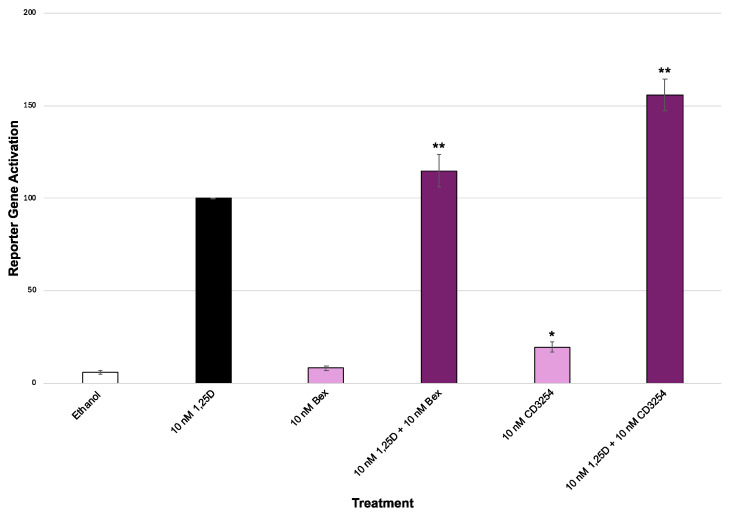
Common VDR polymorphism M1 RXR XDR3 transcriptional activation with exogenous VDR. Bexarotene or **CD3254** was tested via a VDR-RXR (XDR3) VDRE–luciferase-based system in HEK-293 cells with the expression of the M1 polymorphism of VDR. The treatment groups are compared to the positive control of 10 nM 1,25D that was set to 100%, using an ANOVA with post hoc Tukey–Kramer method corrected *t*-tests. All compounds were dosed at 10 nM. An asterisk (*) indicates a statistically significant difference for the rexinoid treatment compared to the ethanol control (*p* < 0.0001). A double asterisk (**) indicates a statistically significant difference between the rexinoid + 1,25D treatment and the 1,25D control (10 nM 1,25D + 10 nM Bex *p* < 0.0014; 10 nm 1,25D + 10nM **CD3254** *p* < 0.0001).

Our results (Figure 5) reveal that rexinoid **CD3254** has greater activity in the context of M1 compared to Bex. In fact, the amount of synergism with **CD3254** using the M1 VDR was 41% greater relative to Bex (Figure 5, bar 4 vs. bar 6; Table 1, 114.9% vs. 155.7%). Moreover, comparing 1,25D and rexinoid plus 1,25D in Figure 5 (M1 VDR), there is a significant difference between 10 nM 1,25D and 10 nM D + Bex (Figure 5, bar 2 vs. 4, *p* < 0.0014), 10 nM 1,25D and 10 nM 1,25D + 10 nM **CD3254** (Figure 5, bar 2 vs. 6, *p* < 0.0001), and 10 nM 1,25D + Bex and 10 nM 1,25D + 10 nM **CD3254** (Figure 5, bar 4 vs. 6, *p* < 0.0001). However, when M4 VDR was used, the amount of synergism of **CD3254** was only 4% greater compared with Bex (Table 1, 139.9% vs. 144.2%).

In directly comparing M1 and M4 VDRs with respect to Bex and **CD3254** synergism, the two polymorphic VDRs do in fact display statistically different (*p* < 0.05) amounts of synergistic activation depending on the rexinoid used. In the presence of the M4 VDR SNP (Table 1, row 2), Bex produced much higher synergism when compared to cells transfected with exogenous M1 VDR. Specifically, with the M4 VDR, the amount of synergism for Bex was 25% greater compared to Bex synergism with M1 VDR (Table 1, 139.9% vs. 114.9%). However, rexinoid **CD3254** produced less synergism with M4 vs. M1 VDR (row 4). Specifically, when using the M4 VDR, the amount of synergism for **CD3254** was 11.5% less compared to the M1 VDR (Table 1, 144.2% vs. 155.7%, *p* < 0.0001).

Also, as observed in Figure 3B and Figure 5, cells treated solely with Bex or **CD3254** (no 1,25D, the primary ligand for VDR) exhibited some degree of activation. In Figure 5 with M1 VDR, comparing ethanol with rexinoid treatments alone, there is a significant difference between ethanol and 10 nM **CD3254** (*p* < 0.0001) and between 10 nM **CD3254** and 10 nM Bex (*p* < 0.0001). In Figure 3B, comparing ethanol with rexinoid treatment alone, there is a significant (*p* < 0.05) difference between ethanol and 10 nM Bex and ethanol and 10 nM **CD3254**, as well as a difference (*p* < 0.05) between 10 nM Bex and 10 nM **CD3254**. These observations suggest that the vitamin D receptor (VDR) may not function exclusively as a non-permissive heterodimer. Furthermore, both M1 (Figure 5) and M4 (Figure 3B) reveal the same behavior concerning a permissive VDR. These findings imply that there is some degree of rexinoid-driven RXR-VDR heterodimerization and activation when the 1,25D ligand is absent and that this occurs with both M1 and M4 VDR.

### 3.4. Vitamin D Concentration and Its Impact on Synergism

Vitamin D deficiency is common in every part of the United States and is reported to be prevalent in between 25% and 75% of adults [49]. The effects of vitamin D concentration in the context of synergism with rexinoids and vitamin D are unknown; therefore, we aimed to explore this question. Our results indicate that the synergism of vitamin D and rexinoids is in fact more robust in the presence of a lower concentration of vitamin D, while the specific rexinoid ligand (analog) shows no difference in synergistic activity.

**Figure 6:**
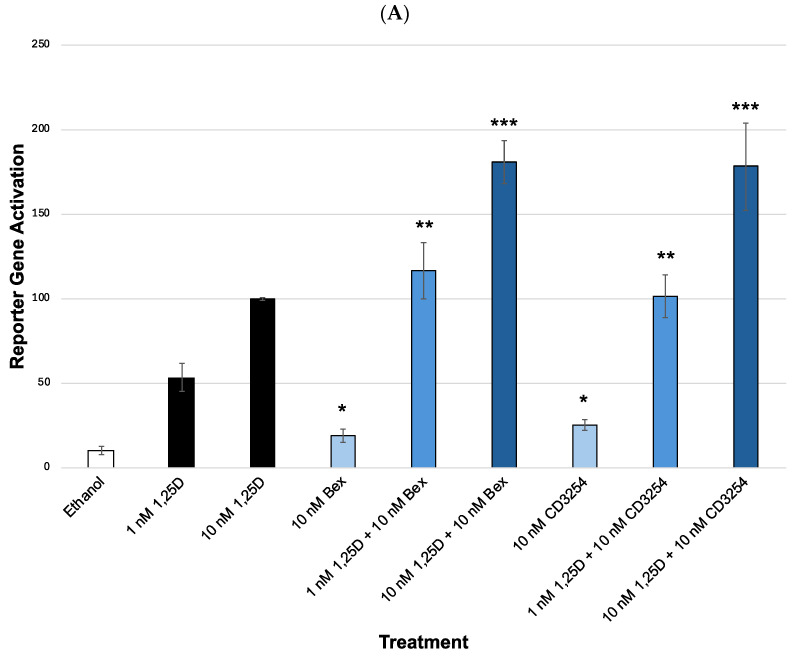

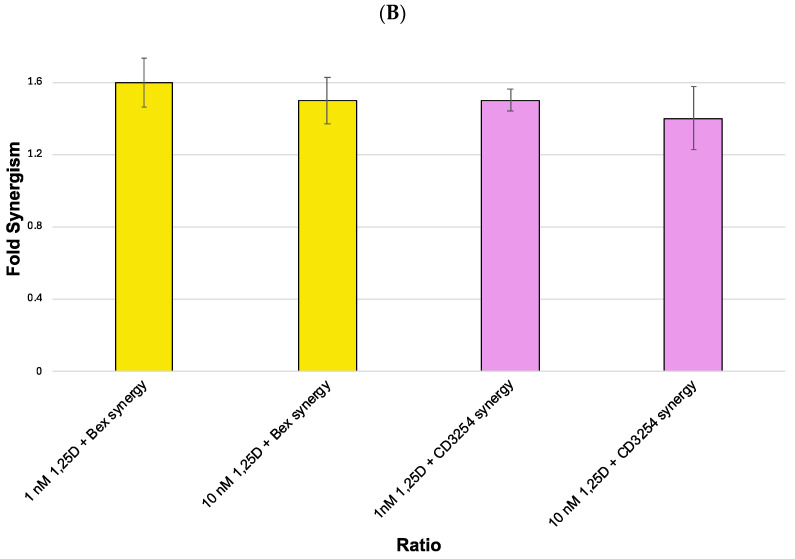
Effect of concentrations of 1,25D on XDR3 VDRE transcriptional activation (endogenous M4 VDR). (**A**) 1,25D concentration assay with bexarotene or **CD3254** at two concentrations of 1,25D, 1 nM and 10 nM. Biological evaluation of 1,25D and bexarotene/**CD3254** via a VDR-RXR (XDR3) VDRE–luciferase-based system in HEK-293 cells. The treatment groups are compared to the positive control 10 nM 1,25D (set to 100%) or to 1 nM 1,25D, using an ANOVA with post hoc Tukey–Kramer method corrected *t*-tests. An asterisk (*) indicates a statistically significant difference between the ethanol control and the 10 nM rexinoid treatment (Bex *p* = 0.0004 and **CD3254** *p* < 0.0001). Double asterisks (**) indicate a statistically significant difference between the 1 nM 1,25D control and 1 nM 1,25D+rexinoid (*p* < 0.0001). Triple asterisks (***) indicate a statistically significant difference between the 10 nM 1,25D control and 10 nM 1,25D+rexinoid (*p* < 0.0001). (**B**) Analysis of fold changes in differing concentrations of 1,25D. A ratio was calculated for VDR activation with vitamin D concentrations compared to their appropriate control group. Yellow bars indicate Bex and pink bars indicate **CD3254**. For synergy, the equation used was ([VDRE activity of rexinoid+1,25D]/[VDRE activity of 1,25D] + [VDRE activity of rexinoid alone]) at each individual 1,25D concentration.

Our results reveal that Bex and **CD3254** show more activity when less vitamin D is present within the cells. Figure 6A tests two varying concentrations of 1,25D: 1 or 10 nM, along with a 10 nM concentration of the rexinoids in the VDR-RXR (XDR3) VDRE–luciferase-based system using HEK-293 cells. The ratio of each 1,25D concentration activity with/without Bex (or analog) is shown in Figure 6B. The ratio graphs (folds) were plotted to compare the synergistic group (with Bex or an analog) with the proper 1,25D (only) control group (since we employed two different 1,25D concentrations).

As shown in Figure 6A, when comparing the synergism of 10 nM 1,25D+Bex to the positive control of 10 nM 1,25D (alone), an increase from 100% activity (1,25D alone) vs. 181% activity with 1,25D+Bex was seen (Figure 6A, comparing bars 3 and 6), while the synergism of 1 nM 1,25D+Bex compared to the positive control of 1 nM 1,25D (alone) resulted in an increase from 57% activity to 116% activity with Bex (Figure 6A, comparing bars 2 and 5). The values in the ratio graph (Figure 6B) indicate that there is a 1.5-fold increase in VDR activity (synergism) at 10 nM 1,25D with Bex (Figure 6B, bar 2) and a higher 1.6-fold increase in VDR synergistic activation at 1 nM 1,25D with Bex (Figure 6B, bar 1).

Comparing the synergism of 10 nM 1,25D+**CD3254** to the positive control of 10 nM 1,25D (alone), the results demonstrate an increase from 100% activity (1,25D alone) vs. 178% activity with **CD3254** (Figure 6A, comparing bars 3 and 9), while the synergism of 1 nM 1,25D+**CD3254** compared to the positive control of 1 nM 1,25D (alone) resulted in an increase from 50% activity to 102% activity with **CD3254** (Figure 6A, comparing bars 2 and 8). The values in the ratio graph (Figure 6B) indicate that there is a 1.4-fold increase in VDR activity (synergism) at 10 nM 1,25D with **CD3254** (Figure 6B, bar 4) and a higher 1.5-fold increase in VDR synergistic activation at 1 nM 1,25D with **CD3254** (Figure 6B, bar 3) as well.

When Bex and **CD3254** are compared, the results demonstrate almost no difference. For instance, both the synergism of 10 nM 1,25D+Bex and 10 nM 1,25D+**CD3254** were at about 1.8-fold (Figure 6B, comparing bars 2 and 4). The 1 nM 1,25D concentration had a similar trend in that the synergism of 1 nM 1,25D+Bex and 1 nM 1,25D+**CD3254** were at about 2.0-fold (Figure 6B, comparing bars 1 and 3).

### 3.5. Mammalian 2-Hybrid Testing for Synergism Source

The role of heterodimer formation, specifically between VDR and RXR, and how heterodimerization might play a role in the mechanism of synergism between rexinoids and vitamin D is not known. We hypothesized that the source of multi-ligand synergism could be generated by the modulation of heterodimerization between RXR and VDR. Furthermore, we also hypothesized that if additional Bex is added into an assay system that contains both VDR and RXR, the assay may reveal increased heterodimerization when both ligands are present, and this could be the “driver” of synergism. Thus, we chose a mammalian two-hybrid (M2H) luciferase assay to assess the effectiveness of RXR-VDR heterodimerization induced by 1,25D, bexarotene, and **CD3254**. Furthermore, the M2H assay is able to examine heterodimerization and/or synergism under different conditions including different concentrations of vitamin D, as well as the use of different rexinoids including Bex and **CD3254**.

Comparing the traditional VDRE system with the M2H system reveals notable differences (comparing Figure 1A,B). In the M2H assay (Figure 1B), RXR and VDR display no DNA binding to the reporter plasmid, therefore requiring the inclusion of an autonomous AD (activation domain) and BD (DNA-binding domain), unlike the VDRE system (Figure 1A). In the M2H framework, RXR is fused with the AD; this then facilitates the recruitment of additional transcription factors and coactivators. Additionally, multiple specific BD binding sites are present in the luciferase reporter plasmid; thus, VDR is fused to the BD domain to specifically direct VDR binding to the BD target DNA sequences (Figure 1B).

**Figure 7:**
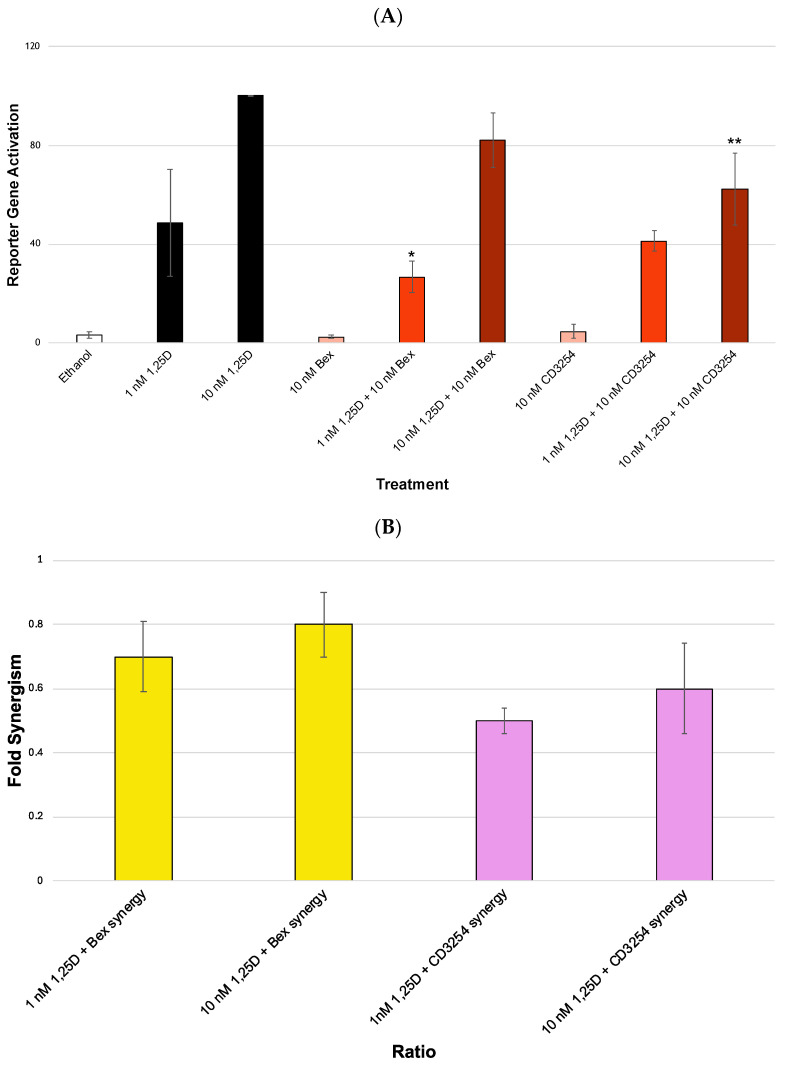
Mammalian 2-hybrid analysis of differing 1,25D concentrations and rexinoids. (**A**) VDR-RXR heterodimerization in a mammalian 2-hybrid system. Biological evaluation of 1,25D, bexarotene, and **CD3254** via a VDR-RXR M2H-based system in HEK-293 cells. The treatment groups are compared to the positive control 10 nM 1,25D (set to 100%) or to 1 nM 1,25D. The treatment groups were compared for their efficacy alone and in combination with 1 nM or 10 nM 1,25D, using an ANOVA with post hoc Tukey–Kramer method corrected *t*-tests. The concentration of the rexinoid treatment groups was 10 nM. An asterisk (*) indicates a statistically significant difference between the 1 nM 1,25D control and 1 nM 1,25D+rexinoid (*p* = 0.0419). Double asterisks (**) indicate a statistically significant difference between the 10 nM 1,25D control and 10 nM 1,25D+rexinoid (*p* = 0.0089). (**B**) Analysis of fold changes in differing concentrations of 1,25D. A ratio was calculated for VDR activation with vitamin D concentrations compared to their appropriate control group. Yellow bars indicate Bex and pink bars indicate **CD3254**. For synergy, the equation used was ([M2H reporter activity of rexinoid+1,25D]/[M2H reporter activity of 1,25D] + [M2H reporter activity of rexinoid alone]) at each individual 1,25D concentration.

Our results demonstrate that when Bex and **CD3254** are added to the M2H system, no synergism is noted. As shown in Figure 7A, when assessing the synergism of 10 nM 1,25D+Bex to the positive control of 10 nM 1,25D (alone), the results demonstrate a decrease from 100% activity vs. 82% activity with 10 nM 1,25D+Bex (Figure 7A, comparing bars 3 and 6, not statistically significant), while the experiment of 1 nM 1,25D+Bex compared to the positive control of 1 nM 1,25D (alone) resulted in a decrease from 54% activity to 27% activity with 1 nM 1,25D+Bex (Figure 7A, comparing bars 2 and 5, *p* < 0.05). There was no significant difference between the ethanol-alone treatment and the rexinoid-alone treatment (Figure 7A, bars 1 vs. 4 vs. 7). The values in the ratio graph (Figure 7B) indicate that there is a 0.8-fold change in VDR activity (actually demonstrating minor anti-synergism) at 10 nM 1,25D (Figure 7B, bar 2) and a 0.7-fold change in the VDR synergistic activation at 1 nM 1,25D with Bex as well (Figure 7B, bar 1).

As shown in Figure 7A, when assessing the synergism of 10 nM 1,25D+**CD3254** to the positive control of 10 nM 1,25D (alone), the results demonstrate a decrease from 100% activity vs. 62% activity with **CD3254** (Figure 7A, comparing bars 3 and 9, *p* < 0.01), while the addition of 1 nM 1,25D+**CD3254** compared to the positive control of 1 nM 1,25D (alone) resulted in a decrease from 54% activity to 41% activity with **CD3254** (Figure 7A, comparing bars 2 and 8). The values in the ratio graph (Figure 7B) indicate that there is a 0.6-fold change in VDR activity (anti-synergism) at 10 nM 1,25D (Figure 7B, bar 4) and a 0.5-fold decrease in the VDR synergistic activation at 1 nM 1,25D with **CD3254** as well (Figure 7B, bar 3).

When Bex and **CD3254** are compared, the results are different. For example, treatment with 10 nM 1,25D+Bex was at about 0.8-fold while the combination of 10 nM 1,25D+**CD3254** was at about 0.6-fold (Figure 7B, comparing bars 2 and 4). The 1 nM 1,25D concentration had a similar trend in that the activity of 1 nM 1,25D+Bex was about 0.8-fold and 1 nM 1,25D+**CD3254** was at about 0.6-fold (compared to 10 nM 1,25D alone) (Figure 7B, comparing bars 1 and 3). Therefore, a difference is observed when Bex is being compared to the analog; however, both show no synergism with vitamin D and perhaps even demonstrate anti-synergism in the context of the mammalian two-hybrid system which assesses the possible contribution of only heterodimerization between VDR and RXR as a driver of two-ligand synergism.

### 3.6. Synergism in U87 Brain Cells

In the previous experiments, we have consistently employed (and observed synergism in) human embryonic kidney cells under different conditions. However, in the final set of experiments, we sought to determine if the synergism assessed in HEK cells can be observed in a different human cellular background, specifically in U87 human glioblastoma cells.

**Figure 8:**
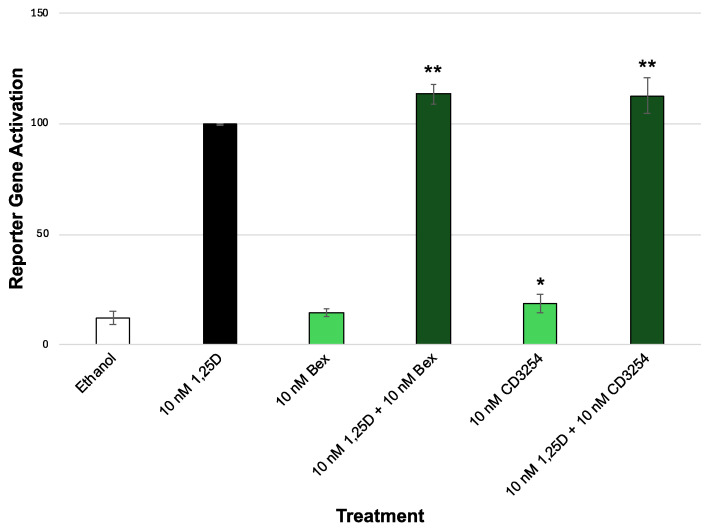
VDR and RXR XDR3 VDRE transcriptional activation in U87 cells with exogenous VDR (M4). Biological evaluation of 1,25D and bexarotene/**CD3254** via a VDR-RXR (XDR3) VDRE–luciferase-based system in U87 cells with the addition of exogenous M4 VDR cDNA. The treatment groups are compared to the positive control 1,25D that was set to 100%, using an ANOVA with post hoc Tukey–Kramer method corrected *t*-tests. All compounds were dosed at 10 nM. An asterisk (*) indicates a statistically significant difference between the ethanol control and the 10 nM rexinoid treatment (*p* = 0.025). Double asterisks (**) indicate a statistically significant difference between the 10 nM 1,25D control versus 10 nM 1,25D+rexinoid (Bex or **CD3254**) (10 nM 1,25D + 10 nM Bex, *p* = 0.0032; 10 nM 1,25D + 10 nM **CD3254**, *p* = 0.0025).

Our results indicate that U87 cells (Figure 8 and Table 2) displayed very modest synergism compared to the HEK-293 cells with overexpressed human VDR (Figure 3B). In HEK cells, the 10 nM 1,25D+Bex treatment resulted in a 40% increase in VDR activity compared to 10 nM 1,25D alone (Figure 3B, comparing bars 2 and 4). In U87 cells, the 10 nM 1,25D+Bex treatment resulted in only a 14% increase in VDR activity compared to 10 nM 1,25D alone (Figure 8, comparing bars 2 and 4). Similarly, with **CD3254**, in HEK cells, the 10 nM 1,25D+**CD3254** treatment resulted in a 44% increase in VDR activity compared to 10 nM 1,25D alone (Figure 3B, comparing bars 2 and 14). In U87 cells, the 10 nM 1,25D+**CD3254** treatment resulted in only a 13% increase in VDR activity compared to 10 nM 1,25D alone (Figure 8, comparing bars 2 and 6). When comparing both the HEK and U87 cell data between Bex and **CD3254**, the amount of synergism in HEK cells was 4% greater with **CD3254** versus Bex (Table 2, 144.2% vs. 139.9%, HEK). In U87 cells, there was virtually no difference between **CD3254** versus Bex (Table 2, 112.7% vs. 113.5%, U87).

Bex and **CD3254** do display a difference in synergistic activity between the HEK and U87 cells when combined with 1,25D. Comparing ethanol with the rexinoid treatment alone, there is a significant difference between ethanol and 10 nM **CD3254** (Figure 8, bar 1 vs. bar 5, *p* = 0.0250). Comparing 1,25D treatment with 1,25D and rexinoid treatment, there is a significant difference between 10 nM 1,25D and 10 nM 1,25D + Bex (Figure 8, bar 2 vs. 4, *p* = 0.0032) and 10 nM 1,25D + 10 nM **CD3254** (Figure 8, bar 2 vs. 6, *p* = 0.0025). In HEK cells, 10 nM 1,25D+Bex produced much higher synergism in HEK-293 cells than in U87 cells (Table 2, row 2, 139.9% vs. 113.5%, *p* = 0.0002). Similarly, the amount of synergism for **CD3254** was 32% greater in HEK cells compared to the U87 cells (Table 2, row 4, 144.2% vs. 112.7%, *p* < 0.0001).

## 4. Discussion

### 4.1. Effect of VDRE DNA Platforms, VDR Concentrations, and RXR Analogs

In the present study, we show that both an RXR and a VDR ligand can activate the VDR-RXR heterodimer (although to a different extent) when human cells are treated with each ligand together or separately. The nature of the VDRE DNA platform had a substantial impact on transcriptional activity that was generated from the RXR-VDR complex because we observed different results when employing two distinct VDREs: XDR3 and PER6. The XDR3-based assay (Figure 3) revealed higher levels of activation than in the PER6-based assays (Figure 4), demonstrating that the DNA element that is targeted can have a significant impact on the activity levels produced by the VDR-RXR heterodimer. If we were to take the results and apply them in a clinical setting, the results suggested that therapeutic strategies might differ based on the targeted DNA element (i.e., the target gene(s)). The XDR3 is composed of two direct repeats of a specific DNA sequence, with a spacer region of three nucleotides, and is mostly found in the promoter of genes involved in calcium homeostasis and bone mineralization. In contrast, PER6 comprises two DNA half-sites positioned in an everted manner relative to each other and is found in genes coding for enzymes that play a role in detoxification processes within the cell.

VDR concentration and level of expression also have an impact on how much synergism is produced from the VDR-RXR heterodimer. The results demonstrate the impact of endogenous VDR (receptor present in the HEK-293 cells naturally) versus exogenous VDR (additionally transfected VDR cDNA) primarily in the XDR3 VDRE (Figure 3). Figure 3A, which contains only endogenous VDR (XDR3), shows much higher ligand-dependent activation and synergism compared to Figure 3B with exogenous (extra) VDR (XDR3). One possible explanation for these results is that the presence of a lower concentration of cellular VDR is more dependent on the VDR and/or RXR ligand to “drive” heterodimerization. In contrast, when a large amount of exogenous/overexpressed VDR is present, this driving force may be diminished because excess VDR can start to heterodimerize with RXR even in the absence of vitamin D or rexinoid; thus, the equilibrium is shifted toward heterodimer formation causing a decrease in ligand-dependent heterodimerization, and thus less “synergism” and resultant activation of transcription. This trend with lower VDR leading to greater synergism between VDR and RXR ligands is not observed in the experiments with endogenous versus exogenous VDR in the PER6 assays (Figure 4), as the PER6 VDRE with endogenous VDR (Figure 4A) never demonstrated higher levels of gene activation as XDR3 VDRE did with endogenous VDR (Figure 3A). This observation with PER6 suggests that the heterodimer affinity for the VDRE (XDR3 vs. PER6) can influence the level of synergism, as well as the effects of receptor concentrations on synergistic activation.

A final insight that is revealed from these results is that the chemical nature of the RXR ligand (rexinoid) has an impact on synergistic activation as well. The results demonstrate that the XDR3 VDRE and endogenous VDR represent the highest synergistic activity. From the experiments containing XDR3 and endogenous VDR (Figure 3A), the analog with the highest synergism is **CD3254**. Therefore, **CD3254** has the potential to be a desirable alternative to Bex in the context of a rexinoid with enhanced synergistic potential. The Bex analogs likely differ in their level of synergism due to their structural differences (Figure 2) and physiochemical differences (see below).

This research demonstrates a proof of concept of utilizing a rexinoid-like **CD3254** with a VDR ligand, and more research would need to be performed to determine the appropriate rexinoid and VDR ligand to treat different diseases. For example, in colon cancer, it has been seen that vitamin D has demonstrated therapeutic potential but may be limited due to VDR mutations [50], and a combinatorial approach of a rexinoid and a VDR ligand could help mitigate any effects of VDR mutations. Additionally, treatment with both RXR and VDR ligands has been shown to decrease oxidative stress and alleviate diabetic atherosclerosis in a mouse model [51]. Finally, circulating vitamin D levels are often very low in CTCL patients, and it has been shown that treating CTCL cells with a combination of vitamin D and Bex can stimulate apoptosis [52]. Thus, perhaps treating RXR-mediated diseases with a combinatorial approach could prove superior to monotherapy.

### 4.2. Effect of VDR Polymorphisms and Lower Levels of Vitamin D

Past studies have suggested that the human VDR containing an f/M1 SNP in the FokI VDR locus, which initiates transcription at an alternative ATG start site, is less effective in mediating VDRE-dependent transcription in comparison to the F/M4 variant. The M1 VDR with 427 amino acids and the M4 VDR with 424 amino acids likely possess a difference in their overall 3D structure which could cause a difference in the function of the protein, with the M4 VDR being more physiologically active. Furthermore, population studies revealed the universality of these isoforms in the human population and demonstrated a ratio of 35% VDR M1 to 65% VDR M4 [31]. Past findings have indicated that the M4 variant is a comparatively newer evolutionary trait that had arisen shortly after the divergence of hominids from apes [31]. The prevalence of the M4 VDR and its more recent divergence suggests its evolutionary advantage compared to the M1 VDR, perhaps due to its more active role in systems such as bone physiology in the face of reduced calcium intake within the human diet [31].

However, this conclusion about the greater activity of the M4 VDR cannot be generalized to the synergism of rexinoids and vitamin D. If this conclusion were true, we would have observed greater synergism by **CD3254** with the M4 VDR compared to **CD3254** synergism with the M1 VDR, but instead, the M1 VDR displayed more synergism (Table 1, row 4). Conversely, with Bex, the level of synergism was greater with M4 vs. M1 (Table 1, row 2). Therefore, in this set of experiments, the results suggest that there are analog-specific polymorphic differences in VDR-RXR synergism, and this unique observation may be of keen interest to personalized medicine [53]. Taken together, the data support the hypothesis that specific SNPs in VDR influence the synergism of rexinoids and vitamin D, which can allow for a personalized approach to the treatment of vitamin D- or rexinoid-related disorders.

The reported global frequency of vitamin D deficiency is anywhere from 15% to 90% depending on the study. In this context, we demonstrated that the synergism between vitamin D and Bex or **CD3254** is actually better when a lower concentration of vitamin D is available (Figure 6B, bars 1 and 3 vs. bars 2 and 4). This can be seen by testing the synergistic activity of Bex or **CD3254** in combination with 1 nM or 10 nM 1,25D. In addition, Bex and **CD3254** treatment showed no significant difference in activation, as both rexinoids were more effective in synergism at lower concentrations of 1,25D. A proposed mechanism for these results may be that when less 1,25D is available, rexinoids compensate for the lack of 1,25D and can more efficiently activate the RXR heterodimeric partner receptor.

### 4.3. Mammalian 2-Hybrid Testing for Mechanism of Action of Synergism

Our results indicate that the source of synergism between 1,25D and rexinoids is likely not due to improved heterodimerization. The results reveal that when Bex is added to the M2H system in the presence of multiple concentrations of 1,25D, this causes an inhibitory response (Figure 7). As described in Figure 1B, the M2H assay only requires the 1,25D ligand to elicit heterodimerization, and once heterodimerization takes place, the addition of the RXR ligand apparently does not further “enhance” heterodimerization or contribute to additional “activation” since the AD of RXR-AD does not require the presence of a rexinoid in order to “activate” transcription of the luciferase gene. In contrast, one possible reason for the inhibition that is observed when both 1,25D and a rexinoid are present simultaneously in the M2H system is that RXR can also homodimerize when bexarotene is added. This RXR-RXR homodimerization pathway leads to competition between RXR-VDR heterodimerization (driven by 1,25D) and RXR-RXR homodimerization (driven by the additional presence of Bex). The result is that while heterodimerization does occur in the presence of only 1,25D, no additional “activity” (synergism) is observed when Bex is added. Indeed, RXR is likely recruited away from the RXR-VDR heterocomplex, therefore ameliorating synergism in the M2H assay. Nonetheless, if heterodimerization was the major driving force for the additional activation (synergism) in the presence of both VDR and RXR ligands, then the M2H system (which is a protein–protein interaction assay) would likely have shown an increase in reporter gene activity as opposed to a slight decrease.

### 4.4. Synergism and Cellular Background

The findings from these experiments indicate that synergism is not observed in human brain glial cells (U87) versus the embryonic kidney system (HEK-293). Therefore, we propose that synergism cannot be generalized to all cell types, and the type of cell line used to attain synergism will therefore impact VDR-RXR synergistic activation. Future experiments should include a wide variety of cell types such as HUT78 cells (a human T-cell lymphoma cell line), breast cancer cells (MCF-7), and a bone cancer cell line (ROS 17/2.8) to further define the relationship between vitamin D and rexinoid coactivation and synergy in a variety of relevant vitamin D and rexinoid target tissues.

Furthermore, the differences in synergism in different cell types such as HEK-293 cells and U87 cells could also be explained by possible cell-specific traits, genetic diversity, cellular environments, disease-related aspects, and/or the differentiation states of the cells. For example, disease-specific factors may influence the occurrence of synergism, as the U87 cells are derived from glioblastoma while HEK-293 cells are derived from embryonic kidney cells. Therefore, the underlying disease state of the U87 includes differences in epigenomic and genetic composition, signaling pathways, gene expression, and metabolism, which can all possibly impact the synergistic effects that were observed in HEK-293 cells. Moreover, HEK-293 cells and U87 cells may also represent different stages of cellular differentiation, since HEK-293 cells derived from human embryonic kidney cells are traditionally less differentiated and display characteristics of undifferentiated cells. In contrast, U87 cells, derived from glioblastoma, may represent a more differentiated state, resembling more mature cells from the brain. Therefore, some of these cellular differences could affect the responsiveness of each cell line to vitamin D and its receptor partners.

### 4.5. Rexinoid Compounds and Physiochemical Characteristics

**Figure 9:**
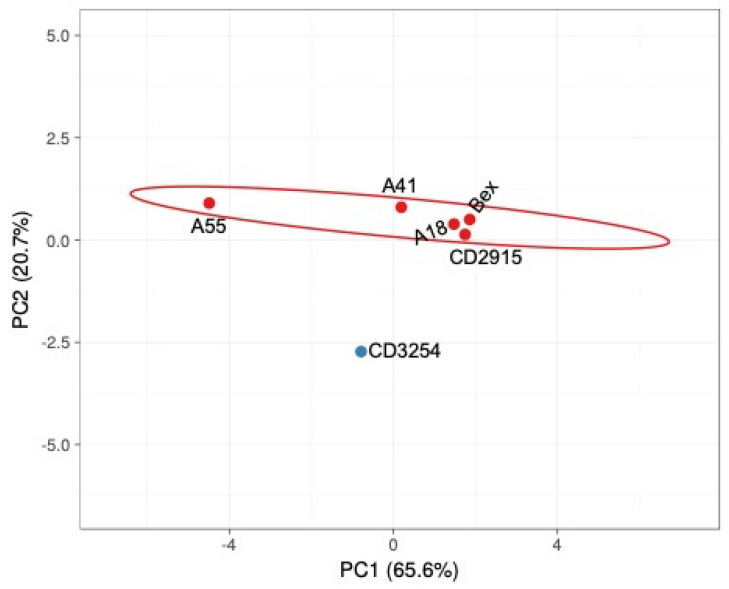
PCA of physiochemical properties of rexinoids used in this study. Properties as outlined in Table 3 were utilized to generate a PCA plot. Unit variance scaling is applied to rows; SVD with imputation is used to calculate principal components. X and Y axes show principal component 1 and principal component 2 which explain 65.6% and 20.7% of the total variance, respectively. Prediction ellipses are such that with a probability of 0.95, a new observation from the same group will fall inside the ellipse. N = 6 data points.

To determine if the physiochemical differences in the compounds are driving the synergy seen in HEK-293 cells, we utilized the characteristics listed in Table 3 for a principal component analysis to identify if these characteristics could be driving the differences identified. As seen in Figure 9, all compounds cluster together with the exception of **CD3254**, which is an outlier on the PCA and also demonstrates the strongest synergism with the classic DR3 vitamin D response element (Figure 3B). This indicates that perhaps the chemical properties of the rexinoids can be exploited to determine the designer binding of compounds to receptors to help individualize therapies. These characteristics and analyses demonstrate only a proof of concept of possibilities, as reported by our group previously [21,35], but could serve as a first step before the synthesis of new rexinoids.

## 5. Conclusions

We hypothesized that RXR ligands such as Bex and other rexinoids and VDR ligands (1,25D) could stimulate a synergistic effect and demonstrate higher transcriptional activity than either ligand alone. Thus, we designed experiments to examine the effect of the treatment of vitamin D and rexinoids on a variety of gene expression constructs to probe the effects of VDR- and RXR-selective ligands on gene expression. Our results demonstrate that in certain cellular contexts, treatment of the cells with the natural ligand for the vitamin D receptor (1,25D) and rexinoid ligands for RXR demonstrates synergy as seen by increased transcription from a classical DR3 VDRE (XDR3) promoter element. We demonstrate that different rexinoids possess different synergistic activities, and these activities are observed more robustly at lower, not higher, vitamin D concentrations. Intriguingly, this synergy does not occur in all cell types or on all VDRE promoter elements, suggesting cell- and pathway-specific fine-tuning of gene expression. Furthermore, VDR-RXR synergism differs depending on the VDR polymorphism type (M1 or M4). We also show that enhanced heterodimer formation is likely not the predominant cause of the effects seen. Moreover, these differences in synergy might be attributable to the physiochemical properties of the ligands. We conclude that the synergism between rexinoids and vitamin D may represent an untapped pathway to explore the feasibility of combinatorial therapeutic treatment strategies since some diseases (e.g., cancers) are thought to be amenable to treatment by both rexinoids and vitamin D. Thus, it may be useful to elucidate the presence of multi-ligand pathways that may facilitate synergistic activation of VDR signaling.

## Figures and Tables

**Table 1:** Comparison of transcriptional activation synergy between rexinoids and vitamin D with common VDR polymorphisms. Cells were treated with bexarotene or **CD3254** in the presence of VDR, either the M1 (ff) or M4 (FF) variant (overexpressed in HEK-293 cells) along with an XDR3 VDRE–luciferase-based reporter plasmid. The mean and standard error for % transactivation (relative to the 1,25D positive control, set to 100%) are shown. All compounds were dosed at 10 nM. Data are taken from Figure 3B for M4 VDR or Figure 5 for M1 VDR. Comparisons were evaluated using an ANOVA with post hoc Bonferroni method corrected *t*-tests. An asterisk (*) indicates a statistically significant difference between M4 and M1 (*p* < 0.0001).

	M4 (FF)	M1 (ff)
10 nM Bex	9.8 (1.0)%	8.3 (1.3)%
10 nm 1,25D + 10 nm Bex *	139.9 (6.3)%	114.9 (8.9)%
10 nM CD3254	16.8 (4.0)%	19.6 (2.8)%
10 nM 1,25D + 10 nM CD3254	144.2 (15.4)%	155.7 (8.6)%

**Table 2:** Comparison of the synergy of rexinoids and vitamin D in HEK-293 and U87 cells. Cells were treated with bexarotene or **CD3254** after transfection with M4 VDR cDNA in HEK-293 or U87 cells. VDR activity was assessed via an XDR3 VDRE–luciferase-based assay and the mean and standard error of luciferase activity are reported. The treatment groups are compared to the positive control of 10 nM 1,25D that was set to 100%, using an ANOVA with post hoc Bonferroni method corrected *t*-tests. All compounds were dosed at 10 nM. Data are taken from Figure 3B (HEK-293) or Figure 8 (U87). An asterisk (*) indicates a statistically significant difference between HEK-293 and U87 results (10 nM 1,25 D + Bex *p* = 0.002; 10 nM 1,25D + 10 nM **CD3254** *p* < 0.0001).

	HEK-293	U87
10 nM Bex	9.8 (1.0)%	14.5 (1.6)%
10 nM 1,25D + 10 nM Bex *	139.9 (6.3)%	113.5 (4.3)%
10 nM CD3254	16.8 (4.0)%	18.7 (4.3)%
10 nM 1,25D + 10 nM CD3254 *	144.2 (15.4)%	112.7 (8.0)%

**Table 3:** Physiochemical properties of bexarotene and rexinoids used in this study, utilized for Figure 9 PCA. MW is the molecular weight. HBD is a hydrogen bond donor. HBA is a hydrogen bond acceptor. cLogP is the octanol–water partition coefficient. Violations are the number of violations of Lipinski’s Rule of Five. cLogD is the distribution coefficient. TPSA is the topological surface area. LogS denotes solubility. Water solubility was scored as 2 for not soluble, 1 for poorly soluble, and 0 for soluble. CNS MPO Score is the Central Nervous System Multiparameter Optimization Score. Calculations are described in Materials and Methods.

Property	Bexarotene	A18	A41	A55	CD2915	CD3254
MW	348.484	366.474	364.45	366.503	348.484	364.483
HBD	1	1	1	1	1	2
HBA	2	2	3	4	2	3
cLogP	5.86	6.12	5.94	3.8596	5.62	5.27
Violations	1	1	1	0	1	1
cLogD	3.835	3.68	3.42	2.67	4.015	3.48
TPSA	37.3	37.3	37.3	53.43	37.3	57.53
Log S	−7.62	−7.8	−7.48	−6.391	−7.17	−6.95
Water Solubility	1	1	1	1	1	1
CNS MPO Score	3.8	3.8	4	4.2	3.7	3.7

## Data Availability

The dataset used is available upon request from the authors.
